# FAMILY CAREGIVER SOCIAL CONNECTEDNESS: TECHNOLOGY USE ACROSS GENERATIONS DURING THE COVID-19 PANDEMIC

**DOI:** 10.1093/geroni/igac059.401

**Published:** 2022-12-20

**Authors:** Janet Pohl, Jude Kolodisner, David Coon

**Affiliations:** Arizona State University, Phoenix, Arizona, United States; Arizona State University, Tempe, Arizona, United States; Arizona State University, Phoenix, Arizona, United States

## Abstract

During the COVID–19 pandemic, maintaining connectedness was difficult for caregivers. Family caregivers represent multiple generations whose experience with and use of social technology to maintain connectedness can vary and differentially impact critical health outcomes. The aims of this study were to examine caregiver connectedness and technology preferences across three generations of caregivers who provide care to older adults with chronic illnesses. The semi-structured focus-groups/interviews conducted in August of 2020 with family caregiver participants including Millennials (n=6), Generation X (n=5), and Boomer (n=8). Two researchers analyzed the transcribed content via thematic analysis. Similarities and differences across generations were assessed via comparative analysis. The themes that emerged from the data were: (1) Millennials (a) Altered stage of life, (b) Altered connectedness, (c) Need others to understand, (d) Stay away from social network sites; (2) Generation X (a) Altered connectedness, (b) Need others to understand, (c) Burden, (d) Fear-of-failure; and (3) Baby Boomer (a) Altered connectedness, (b) Technology builds connectedness, (c) Information seeking. All generations expressed alterations in connectedness with caregiver role. Millennials and Generation X caregivers emphasized need for others to understand that caregiving altered their lives with unique responsibilities. Technology use differed across the generations, with Millennial texting for confidential communications. Millennial and Generation X caregivers do not use social media due to envy of others’ fun. Baby Boomers expressed increased connectedness with the use of Zoom. Understanding the variation in the experience of caregiver connectedness and technology use by generation may identify targets for future caregiver connectedness intervention studies.

